# Feasibility of Rainbow Trout (*Oncorhynchus mykiss*) Fry Rearing in Biofloc System: Effect of Total Suspended Solid Levels on Zootechnical Performance, Intestinal Condition and Antioxidant Enzyme Activity

**DOI:** 10.3390/ani16030446

**Published:** 2026-02-01

**Authors:** Fernanda Regina Delziovo, Mariana Bender, Nataly Oliveira Dos Santos Neves, Larissa Stockhausen, Maria Luiza Silva, Everton Skoronski, Enric Gisbert, Thiago El Hadi Perez Fabregat

**Affiliations:** 1 Departamento de Produção Animal e Alimentos, Centro de Ciências Agroveterinárias, Universidade do Estado de Santa Catarina, UDESC, Av. Luiz de Camões, 2090, Bairro Conta Dinheiro, Lages 88520-000, SC, Brazillari.stock@gmail.com (L.S.); everton.skoronski@udesc.br (E.S.); 2 Aquaculture Program, Institute of Agrifood Research and Technology (IRTA-La Ràpita), Crta, de Poblenou km 5.5, 43450 Sant Carles de la Ràpita, TGN, Spain; enric.gisbert@irta.cat

**Keywords:** growth, salmonid, total suspended solids, sustainability

## Abstract

This study evaluated the effects of different total suspended solid (SST) levels (0, 250 and 350 mg L^−1^) on the productive performance, intestinal condition and antioxidant enzyme activity of rainbow trout (*Oncorhynchus mykiss*) fry reared in a biofloc technology (BFT) system. The experimental design was completely randomized with three treatments and five replicates. A total of 225 rainbow trout fry (0.81 ± 0.06 g) were distributed into 15 tanks (70 L working volume) at a stocking density of 15 fish per tank. Tanks designated for the control treatment (clear water) were connected to a recirculating aquaculture system, whereas BFT treatments were maintained in independent units. Performance analyses were conducted and fish were sampled for intestinal histomorphometry, intestinal microbial counting and antioxidant enzymatic activity. Rearing rainbow trout fry in BFT systems with TSS levels up to 250 mg L^−1^ proved feasible and preserved zootechnical performance, intestinal condition and antioxidant enzyme activity during the early stages of development. The higher TSS concentration negatively affected fish growth performance. However, increased colonization by lactic acid bacteria was observed, indicating that biofloc may serve as a source of probiotic bacteria. The BFT system is a viable alternative for rearing rainbow trout fry, enhancing biosecurity and reducing water consumption.

## Simple Summary

This study evaluated the effects of different biofloc total suspended solid (SST) levels (0, 250 and 350 mg L^−1^) on the productive performance, intestinal condition and antioxidant enzyme activity of rainbow trout (*Oncorhynchus mykiss*) fry reared in a biofloc technology (BFT) system. Rearing rainbow trout fry in a BFT system with TSS levels up to 250 mg L^−1^ proved feasible, maintaining zootechnical performance, intestinal condition and antioxidant enzyme activity during the early stages of development. In contrast, the TSS concentration of 350 mg L^−1^ negatively affected fry growth performance. Despite this, increased gut colonization by lactic acid bacteria was observed, indicating that biofloc may serve as a source of probiotic bacteria for rainbow trout. The BFT system is recommended as an alternative system for rearing rainbow trout fry, offering enhanced biosecurity and reduced water consumption.

## 1. Introduction

Rainbow trout (*Oncorhynchus mykiss*) is a carnivorous fish that inhabits clear cold waters [1]. Its fish meat is highly appreciated, presenting a strong market demand. Therefore, it is one of the most produced fish in world aquaculture [2] with an average annual growth rate of 2.5% over the last decade [3]. According to the most recent data [2], inland rainbow trout production reached 739,500 tons, accounting for 1.5% of total global aquaculture. Traditional trout farming relies on flow-through raceway systems that require the use of vast water volumes and specific topographical conditions [4,5,6]. Furthermore, these flow-through systems are susceptible to the introduction of external pathogens and unwanted factors into the farming environment, which can be a biosafety issue [7,8]. Given increasing concerns regarding water scarcity [9], there is a need for more sustainable and environmentally responsible practices in trout farming. In this context, trout production in a biofloc technology (BFT) system emerges as a key promising alternative.

The BFT systems enable intensive fish production with minimal water exchange by using carbon sources to stimulate nitrogen cycling and maintain water quality [10]. During this process, biofloc aggregates are formed, comprising microorganisms and organic residues such as zooplankton and phytoplankton [11]. Furthermore, these particles are rich in protein, lipids, vitamins and micronutrients [12], making them a possible supplementary feed source for farmed animals [13]. Flocs may also be a source of beneficial probiotic bacteria with benefits to the intestine [14]. On the other hand, some factors can negatively impact the health of fish in BFT systems. Decomposition of organic matter, such as the oxidation of ammonia into nitrite, is an oxidative process that can generate reactive oxygen species (ROS) [15]. To counteract oxidative stress, fish rely on the production of antioxidant enzymes [16]. However, during early development, fish display intensified growth and high metabolic activity, leading to mitochondrial working at the maximum rate and increasing the generation of ROS [4]. Several aquatic species have already been successfully produced in BFT systems, though mainly omnivorous organisms, such as *Oreochromis niloticus* [17], *Rhamdia quelen* [18], *Pimephales promelas* [19] and *Cyprinus carpio* [20]. More recently, the feasibility of rearing carnivorous fish, such as *Salminus brasiliensis* and *Arapaima gigas,* was also demonstrated [21,22]. Nonetheless, there remains a demand for studies focusing on the fry and early stages, which are more sensitive and require more stringent rearing control.

Despite its potential, the BFT system has not yet been evaluated for rainbow trout production. The accumulation of total suspended solids (TSS) in the BFT system can be problematic, as excessive concentrations may cause gill damage, impairing gas exchange and osmoregulation [23]. For efficient nitrogen cycling without compromising productive performance, the recommended total suspended solid level range is from 200 to 600 mg L^−1^ [23,24]. In contrast, tolerance limits for salmonids in flow-through systems are significantly lower, around 25 mg L^−1^ [25]. However, previous studies have demonstrated that juvenile rainbow trout reared in closed systems can tolerate higher concentrations due to differences in the physicochemical characteristics of the TSS [26]. We hypothesized that rainbow trout fry can adapt to BFT system rearing and tolerate up to 350 mg L^−1^ of TSS without adverse effects on their productive performance. As an alternative production system, the BFT system may offer both economic and environmental benefits, increasing the sustainability of the activity. Furthermore, possible ingestion of bioflocs may improve nutrient absorption and be a source of probiotics to increase intestinal health [27]. Thus, this study aimed to evaluate the effect of different concentration of total suspended solids in the BFT system on the productive performance, intestinal condition and oxidative enzyme response of rainbow trout fry, as well as validating the feasibility of BFT for early life stages of this salmonid species.

## 2. Methodology

### 2.1. Experimental Design

This study was approved by the Ethics Committee on Animal Use (CEUA) of UDESC with the number 9440250324 on 30 August 2024. The experimental design was completely randomized with three treatments with five replicates. A recirculating aquaculture system RAS served as a control treatment, while two levels of total suspended solids (250 and 350 mg L^−1^) were evaluated in the BFT system. The 56-day experiment was conducted at the Fish Farming Laboratory of the State University of Santa Catarina (UDESC) located in Lages, SC, Brazil.

### 2.2. Animals and Facilities

Rainbow trout fry were purchased from a commercial hatchery (Recanto das Trutas, Bocaina do Sul, SC, Brazil) and acclimated to laboratory conditions for 30 days. After the acclimatization period, fish with an initial average body weight of 0.80 ± 0.06 g (mean ± standard deviation) were randomly distributed into 15 circular tanks (70 L working volume) at the initial density of 15 fish per tank (0.21 fish L^−1^). This value is below the recommendations for rainbow trout farming, which would be at least 13 fish L^−1^ [1]. A lower density was selected due to technical facility limitations and to comply with the Animal Ethics Committee’s guidelines to minimize the number of experimental animals and meet the principles of the 3Rs in animal experimentation.

Control tanks were integrated into a RAS with a UV system, a mechanical and biological filter and individual aeration via microporous hoses. The RAS was selected over the flow-through system as a control group due to the precise control of the experimental conditions. BFT tanks were equipped only with an intensive aeration system with microporous hoses to maintain floc in suspension. Fish were fed a commercial diet (Laguna Carnívoros Crescimento, ADM, Primavera do Leste, MT, Brazil) containing 45% crude protein, 4435 kcal kg^−1^ of gross energy and crude lipid levels of 12% (data provided by feed manufacturer). The feed was ground and sieved to obtain particles of approximately 2 mm in diameter, a size suitable for the mouths of the fish. Feeding was performed twice daily, at 09:00 am and 05:00 pm, to apparent satiety. Since the feed pellets were crushed, they lost their buoyancy. During feeding, the aeration system was temporarily suspended for about 10 min so the fish could identify the pellets entering the water. Since the turbid BFT system made visualization of the ingestion difficult, this technique was necessary for the fish to learn to feed on the surface before the pellets sank, thus allowing for a quantification of satiety. Apparent satiety was considered reached when the fish no longer ascended into the water column to capture the pellets.

Two months prior to the trial, 20 L of matured biofloc inoculum previously cultured with *Oreochromis niloticus* were added to each BFT tank. Clear water from an artesian well was added until the working volume was reached. The same commercial feed used during the experiment was used as a nitrogen source. Molasses (38% carbon) was added to maintain a C–N ratio of 20:1 [28] in each BFT tank from the experiment, assuming that 75% of total ammonia nitrogen from the feed is released into the water [29] to maturate the system. Water quality was monitored, and when nitrate levels began to rise, indicating established nitrification, the system was considered mature and the fish were introduced. Throughout the experimental period, the addition of molasses was not necessary because, due to the use of mature biofloc, the concentrations of total ammonia nitrogen and nitrite were maintained consistently low and stable. When the total suspended solids concentration exceeded the predetermined treatment thresholds (250 and 350 mg L^−1^), as measured using an Imhoff cone, clarification was performed to maintain the levels within the treatment specifications. Water from an artesian well was used in the RAS as well. In both systems, low concentrations of sodium chloride were also used (4–6 ppt) as a prophylactic measure [30].

### 2.3. Water Quality

Water parameters were evaluated weekly (Table 1) and maintained within the optimal range for the rainbow trout. Temperature, pH, salinity and dissolved oxygen were measured using a multiparameter device (AK88, AKSO^®^ Produtos Eletrônicos, São Leopoldo, RS, Brazil). There was a temperature discrepancy between systems due to RAS operational heat generation, maintaining it 2 °C higher than the BFT systems’ tanks. Photoperiod was standardized across treatments (12 h light, 12 h dark). Total suspended solids were determined according to the method described by Rice et al. [31]. Total ammonia nitrogen, nitrite and nitrate levels were measured using a spectrometer (Spectro kit, Alfakit, Florianópolis, SC, Brazil) according to the method of Rice et al. [31]. There were no ammonia peaks, and the presence of nitrate indicated complete nitrogen cycling in the systems. Settleable solids were measured twice a week following the adapted settleable solids methodology, using an Imhoff cone [32]. One liter of water was collected in the cone and allowed to settle for 10 min. Partial water exchanges via sedimentation were performed when the levels of settleable solids exceeded the established thresholds (20 and 30 mL L^−1^). Accumulated solids in the RAS tanks were removed weekly by siphoning.

### 2.4. Fish Performance

At the beginning (7 September 2024) and end (2 November 2024) of the 56-day experiment, the fish were weighed on an analytical balance (Marte; AY220; Santa Rita do Sapucaí, MG, Brazil) to monitor fish growth. Prior to weighing, all fish were submitted to a 24 h fasting period, anesthetized with eugenol at a dose of 60 mg L^−1^ [33] and individually weighed. Productive performance was carried out by analyzing the following parameters:

(1) Weight gain (WG,g)=final average weight−initial average

(2) $$Specific\;growth\;rate\;({\%\;day}^{-1})=\frac{Ln\left(\frac{final\;average\;weight}{initial\;average\;weight}\right)}{experimental\;period}\times 10$$

(3) $$Feed\;intake\;(FI,g)=\frac{total\;dry\;feed\;intake}{number\;of\;surviving\;fish}$$

(4) $$Feed\;conversion\;ratio\;(FCR)=\frac{feed\;intake}{total\;weight\;gain}$$

(5) $$Survival\;rate\;\left(S,\%\right)=\frac{total\;fish\;at\;the\;end}{total\;fish\;at\;the\;beginning}\times 100$$

Two fish per tank (*n* = 10 per treatment) were anesthetized (eugenol at a dose of 60 mg L^−1^) [33] and euthanized by spinal sectioning for intestinal microorganism counting and intestinal histomorphometry. Additionally, eight fish per treatment were sampled for antioxidant enzymes. Intestinal samples destined for enzymatic analysis were immediately stored at −80 °C and subsequently lyophilized (Terroni; Liofilizador LS, São Paulo, Brazil). In contrast, the samples reserved for antioxidant enzymes were lyophilized and stored in a dry place until the analysis was performed at IRTA (Spain) research facilities.

### 2.5. Intestinal Microorganism Counting

Intestines were carefully aseptically removed, weighed and homogenized. The homogenized tissue was diluted (1:10) in tubes containing sterile saline solution (0.65%). The diluted homogenate was plated on Petri dishes with MRS agar (de Man, Rogosa and Sharpe) for lactic acid bacteria (LAB) quantification, TSA agar (tryptic soy agar) for total heterotrophic bacteria quantification and TCBS agar for *Vibrio* sp. Bacterial quantification was performed as described by Kaktcham et al. [34]. Colony-forming units (CFUs) were counted after incubation under optimized conditions (TSA and TCBS media were incubated at 35 °C for 24 h, while MRS medium was incubated at 35 °C for 48 h).

### 2.6. Intestinal Histomorphometry

Sections of approximately 5 cm from the proximal intestine were carefully collected and immediately fixed in 10% buffered formalin for 24 h to prevent tissue degradation. After fixation, the samples were dehydrated in a graded ethanol series, cleared in xylene and embedded in paraffin for sectioning. Thin tissue sections (5 μm thick) were prepared using a microtome according to the methodology adapted from [35]. Samples (2 slides per fish) were stained using the periodic acid-Schiff (PAS) method to highlight structural components and goblet cells [35]. The slides were examined under an optical microscope (Opticam Microscopy Technology, 10× magnification; São Paulo, Brazil) and with a digital camera (Moticam 2300, 3 MP, resolution of 3264 × 2448, Motic, Xiamen, China). One histological section per slide was selected for morphometric analysis. The total height and width of the villi were measured using ToupTek ToupView—x64 image analysis software version 3.7. Goblet cells were quantified in five representative villi per section and were examined per slide.

### 2.7. Antioxidant Enzyme Activity

Prior to analysis, the intestines were sectioned into small fragments and diluted in phosphate buffer 100 mM (pH 7.4) (1:20, *w*/*v*). Samples were homogenized using a Turrax homogenizer (IKA, Staufen, Germany) (in ice bath to prevent thermal degradation), and after that, put into a sonicator for 30 s (pulses of 5 s on and 1 s off) to ensure complete cell lysis. The resulting homogenates were centrifuged at 10,000 rpm for 30 min at 4 °C. The supernatants were then collected and stored for enzymatic activity determination.

Total antioxidant capacity (TAC) was determined using a commercial kit (Sigma-Aldrich; Darmstadt, Germany), based on the reduction in Cu^+2^ to Cu^+^, with absorbance measured at λ = 570 nm [36]. Total glutathione Peroxidase (GPx; EC 1.11.1.9) activity was measured at λ = 340 nm, and it was determined by the NADPH oxidation [37] using glutathione 75 mM and NADPH 8.75 mM as a substrate (e = 6.22 mM^–1^ cm^–1^). Glutathione reductase (GR, E.C. 1.8.1.7) activity was determined by measuring NADPH oxidation at λ = 340 nm using glutathione disulfide 20 mM and NADPH 2 mM as substrate [38]. Superoxide dismutase (SOD, E.C. 1.15.1.1) activity was measured at λ = 450 nm by assessing the inhibition of cytochrome c reduction by O_2_ generated by the xanthine oxidase/hypoxanthine system using a commercial kit (19160 SOD determination kit Sigma-Aldrich; Darmstadt, Germany) [39]. Soluble protein in tissue homogenates was determined using Bradford’s method using bovine serum albumin as a standard [40].

Enzyme activities were expressed as specific activity: nmol substrate catalyzed per minute per mg of protein (nmol min^−1^ mg^−1^ protein^−1^) for GPx and GR. Superoxide dismutase (SOD) activity was expressed as the percentage of inhibition, and total antioxidant capacity (TAC) was expressed in μM for TAC. All assays were performed in triplicate (methodological replicates) at 25 °C using a spectrophotometer (TecanTM infinite M200, Tecan, Männedorf, Switzerland).

### 2.8. Statistical Analyses

For the productive performance analyses, each tank was considered an experimental replicate. For the remaining variables, individual fish were used as replicates. The data were expressed as the mean ± standard deviation (SD). Statistical analyses were performed using SAS^®^ software (SAS Institute Inc., Cary, NC, USA), version 9.4. All the data were subjected to testing to verify the normality of errors, with the Shapiro–Wilk, and the homoscedasticity of variances, using Levene’s test. When normality and homoscedasticity were verified, the differences between the results were determined with analysis of variance ANOVA, and Tukey’s test was used at *p*< 0.05 probability. When the normality was not met, the treatments were compared using the non-parametric Kruskal–Wallis test (*p* < 0.05).

## 3. Results

Fish Performance. At the end of the trial, rainbow trout fry from the BFT 350 mg L^−1^ treatment showed lower (*p* < 0.05) final weight and weight gain compared to the control treatment RAS (Table 2). However, feed intake, feed conversion and survival were not affected (*p* > 0.05) regardless of the production systems and levels of suspended solids in the BFT tanks. Biofloc ingestion was not quantified; however, its presence in the stomachs of fish reared in the BFT system was qualitatively confirmed.

### 3.1. Intestinal Microorganism Counting

Results in terms of counting viable heterotrophic, LAB and *Vibrio *sp. bacteria are shown in Table 3. The BFT350 treatment increased (*p* < 0.05) the concentration of lactic acid bacteria in the intestines of rainbow trout fry (Table 3). Heterotrophic bacteria and *Vibrio* sp. concentration were not affected (*p* > 0.05) regardless of the production system considered (RAS or BFT).

### 3.2. Intestinal Histomorphometry

In the RAS group, the intestinal mucosa of rainbow trout displayed the typical architecture, with well-defined, elongated and uniformly arranged intestinal folds. A continuous epithelial lining was visible along the folds, and goblet cells were regularly distributed. The *lamina propria* appeared thin, without evident thickening or disorganization. The submucosa and muscular layers were distinguishable as intact and well-defined structures, maintaining the expected arrangement. Overall, tissue integrity was preserved, with no detectable structural alterations at this magnification. No significant differences (*p* > 0.05) were observed among treatments for the intestinal histomorphometry of rainbow trout fry regarding villi height and width (Table 4). In contrast, fry from the BFT 350 group exhibited a significantlyly lower count of goblet cells (*p* < 0.05) compared to the RAS group, while BFT250 treatment showed intermediate values.

### 3.3. Antioxidant Enzyme Activity

The levels of total antioxidant capacity (TAC) and activity of antioxidant enzymes did not show any difference (*p* > 0.05) between the treatments (Table 5).

## 4. Discussion

Results from the current study indicated that the use of concentrations up to 250 mg L^−1^ of total suspended solids in the BFT system did not affect the productive performance of rainbow trout fry. To our knowledge, this is the first study to demonstrate the feasibility of utilizing the BFT system for the production of this species. The total suspended solids level of 250 mg L^−1^ was sufficient to maintain adequate water quality, results that were in accordance with those used in the literature for different fish species [23,41]. The biofloc system reduces the water exchange at a minimal rate and can contribute to the sustainability of trout farming. In the present study, water consumption was not measured in the different systems for rainbow trout. However, for Nile tilapia farming in a BFT system, the minimum daily exchange rate is 5% [42]. In contrast, conventional raceway rainbow trout farming systems require much higher turnover rates, with a total water exchange every hour [43]. Excessive amounts of total suspended solids reduce water transparency, compromise feed detection capabilities and reduce feed efficiency and growth rates [44]. Furthermore, TSS accumulation on the gill surface may also hinder oxygen uptake and trigger a physiological stress response [44,45,46]. The recommended concentration of total suspended solids for rainbow trout fry, extrapolated from other salmonids, is 25 mg L^−1^ [47]. However, it has already been demonstrated that rainbow trout juveniles tolerate higher levels of total suspended solids in the RAS system, achieving 71 mg L^−1^ [26]. According to the authors, total suspended solids found in RAS are predominantly of organic origin (i.e., fish feces, rest of uneaten feed pellets) and less abrasive, potentially causing less damage to the gills [26]. Since the solids in the BFT system have similar organic composition and physical characteristics [12,48], these findings support a greater tolerance to higher solids levels than previously reported, like the one shown in the present study.

Under current experimental conditions, the higher concentration of total suspended solids (350 mg L^−1^) negatively affected the growth of rainbow trout fry. For other carnivorous fish species reared in BFT systems, total suspended solids were not measured for validation and results are restricted to juveniles [21,22,49]. In contrast, omnivorous species typically tolerate TSS loads, such as Nile tilapia fry (200–600 mg L^−1^) [24], Prussian carp fry (600–800 mg L^−1^) [50] and red-bellied pacu fry (200–300 mg L^−1^) [45]. The adverse effects of excessive TSS loads may be associated with stress and metabolic changes such as hepatic inflammation and higher cortisol levels [49]. Furthermore, in BFT systems, high concentrations of total suspended solids are linked to decreased visibility and reduced feed intake potential, which can impair growth performance and feed conversion efficiency [51]. Rainbow trout fry are visual feeders [52,53], and managing TSS is critical for maintaining feed visibility and fish performance. Therefore, the impact of TSS on ingestion may have been further amplified by using ground feed, which exhibited reduced buoyancy.

Lactic Acid bacteria are a recognized probiotic that modulate intestinal health and are frequently identified in BFT systems [54,55]. There was an increase in the lactic acid bacteria concentration in the BFT350 treatment. Fish can ingest biofloc and use it as a food source and supply of bioactive compounds [51]. Bioflocs were observed within the digestive tract of rainbow trout fry, confirming ingestion, suggesting these TSS as a potential source of bioactive compounds and probiotics. Lactic acid bacteria have the ability to acidify intestinal pH by producing organic acids, thereby modulating the microbiota [56]. This group of bacteria can synthesize bioactive compounds like bacteriocins and peptides, which can inhibit the growth of other bacteria and potential pathogens [57,58]. Probiotic bacteria can also lead to improved feed conversion [55]. However, the ingestion of bioflocs did not lead to an enhancement of growth performance in rainbow trout fry. The impaired growth performance in the BFT350 treatment may have masked the potential benefits in terms of feed efficiency parameters. No significant differences were detected in *Vibrio* sp. counts, but no count was observed in the BFT250 treatment, indicating a possible modulation of pathogenic microorganisms by the BFT system when 250 mg L^−1^ of total suspended solids were used.

Rainbow trout rearing in the BFT system did not affect the height and width of the intestinal villi. The probiotic bacteria present in the BFT system can lead to improvements in the villi characteristics [59]. In other species, such as Nile tilapia [59] and thinlip gray mullet [60], the improvement of these characteristics has been observed under BFT conditions. High variability in the data may have hindered the detection of differences. Also, it is already known that intestinal histomorphometry does not always show significant changes when microbial-based strategies (e.g., probiotics or biofloc) are applied [61]; it can have masked the beneficial effects of beneficial bacteria. Furthermore, in the BFT350 treatment, there was a reduction in goblet cells in the intestinal epithelium. Goblet cells play a role in mucus secretion and intestinal immunity [62], while such changes in goblet cell density may be related to poor growth performance in this treatment.

BFT system rearing did not alter the antioxidant enzyme activity in the intestine of rainbow trout fry. Antioxidant enzymes play a role against oxidative stress and may also be used as a welfare indicator [12]. Studies show that tissues with higher mitochondrial density and elevated aerobic metabolic capacity have an increase in ROS generation and oxidative damage [63]. Similarly, during early development, fish display intensified growth and high metabolic activity, leading to mitochondria working at the maximum rate and increasing the generation of ROS. In response to ROS accumulation, the antioxidant system activates specific enzymes to neutralize these radicals, preventing cellular damage or apoptosis [16]. The absence of differences between treatments suggests that the BFT system did not affect the intestinal antioxidant response of the fish. This demonstrates that the BFT system is safe for rainbow trout with no indication of compromised welfare. Notably, even in the BFT350 treatment, which showed the lowest growth performance, the activity of antioxidant enzymes was not affected.

## 5. Conclusions

Rearing rainbow trout fry in BFT systems with total suspended solid levels of up to 250 mg L^−1^ was feasible as it maintained zootechnical performance, intestinal condition and antioxidant enzyme activity during the early development stages. Conversely, a concentration of 350 mg L^−1^ negatively affected fish growth. Despite the reduced growth at higher solid levels, the increased colonization by lactic acid bacteria suggests that biofloc serves as a source of probiotic bacteria for rainbow trout. Therefore, BFT systems managed with TSS levels of up to 250 mg L^−1^ are recommended as a viable alternative for rearing rainbow trout fry, offering enhanced biosecurity and water saving.

## Figures and Tables

**Table 1:** Water quality analysis values for rainbow trout after 56 days of the experiment.

	RAS	BFT 250	BFT 350
Temperature (T°C)	20.9 ± 1.53	18.7 ± 0.96	18.8 ± 0.97
Total suspended solids (mg L^−1^)	4.10 ± 1.55	274.72 ± 31.34	365.35 ± 36.67
Settleable solids (mL L ^−1^)	0.00 ± 0.00	24.32 ± 4.40	33.38 ± 4.03
Ammonia (mg L^−1^)	0.03 ± 0.08	0.00 ± 0.00	0.00 ± 0.00
Nitrite (mg L^−1^)	0.07 ± 0.12	0.00 ± 0.03	0.00 ± 0.00
Nitrate (mg L^−1^)	64.77 ± 47.83	228.8 ± 31.6	327.6 ± 50.2
pH	8.89 ± 0.10	8.82 ± 0.91	8.81 ± 0.10
O_2_ (mg L ^−1^)	7.11 ± 0.73	6.87 ± 0.67	6.91 ± 0.62
Salinity (mg L ^−1^)	4.42 ± 0.77	6.38 ± 1.27	6.14 ± 0.95

RAS: recirculation aquaculture system; BFT250: biofloc 250 mg L^−1^; BFT350: biofloc 350 mg L^−1.^

**Table 2:** Average values (±standard deviation; n = 5) of performance variables of rainbow trout (*Oncorhynchus mykiss*) fry reared in different levels of suspended solids in water after 56 days.

	RAS	BFT 250	BFT 350	*p* Values
Initial Weight (g)	0.83 ± 0.05	0.77 ± 0.02	0.84 ± 0.06	0.079
Final Weight (g)	3.13 ± 0.18 ^a^	2.74 ± 0.26 ^ab^	2.41 ± 0.49 ^b^	0.017
Weight Gain (g)	2.29 ± 0.19 ^a^	1.98 ± 0.24 ^ab^	1.57 ± 0.54 ^b^	0.024
Specific growth rate (% day ^−1^)	2.36 ± 0.14 ^a^	2.34 ± 0.10 ^ab^	1.85 ± 0.47 ^b^	0.041
Feed Intake (g fish^−1^)	2.86 ± 0.80	3.06 ± 0.74	3.11 ± 0.54	0.855
Feed Conversion ratio	1.34 ± 0.12	1.55 ± 0.39	1.98 ± 0.65	0.166
Survival (%)	89.33 ± 7.60	88.33 ± 11.39	78.33 ± 15.75	0.336

Different letters in the same row indicate a significant difference (*p* < 0.05) within the factors (biofloc concentration levels) by the Tukey test. RAS: recirculation aquaculture system; BFT250: biofloc 250 mg L^−1^; BFT350: biofloc 350 mg L ^−1^.

**Table 3:** Average values (±standard deviation; n = 5) of intestinal counts of heterotrophic bacteria, lactic acid bacteria and *Vibrio* spp. (log_10_ CFU g^−1^) in rainbow trout (*Oncorhynchus mykiss*) fry reared under different levels of suspended solids in the water after 56 days.

	RAS	BFT 250	BFT 350	*p* Values
Heterotrophic	7.19 ± 0.65	7.28 ± 0.77	6.40 ± 0.88	0.202
Lactic Acid bacteria *	4.0 ± 0.08 ^b^	3.84 ± 0.13 ^b^	4.86 ± 0.33 ^a^	0.001
*Vibrio* sp. **	4.35 ± 0.70	ND	3.95 ± 0.88	0.201

* Means followed by different letters differ (*p* < 0.05) by the Tukey test. ** Treatments do not differ (*p* < 0.05) by Kruskal–Wallis test. RAS: recirculation aquaculture System; BFT250: biofloc 250 mg L^−1^; BFT350: biofloc 350 mg L ^−1;^ ND: not detected.

**Table 4:** Average values (±standard deviation; n = 5) of histological analysis of intestinal villi of rainbow trout (*Oncorhynchus mykiss*) fry reared under different levels of suspended solids in the water after 56 days.

	RAS	BFT250	BFT350	*p* Values
Height (μm)	211.73 ± 43.29	221.51 ± 29.43	182.81 ± 23.32	0.332
Width (μm)	94.50 ± 19.54	82.81 ± 4.28	74.94 ± 14.52	0.185
Goblet Cells (nb)	18.42 ± 3.19 ^a^	18.94 ± 5.96 ^ab^	12.39 ± 3.72 ^b^	0.031

Means followed by different letters differ (*p* < 0.05) by the Tukey test. RAS: recirculation aquaculture system; BFT250: biofloc 250 mg L^−1^; BFT350: biofloc 350 mg L ^−1^; each goblet cell was contacted individually, and the value is expressed per villus.

**Table 5:** Average values of antioxidant enzyme analysis (±standard deviation; n = 21) of the intestines of rainbow trout (*Oncorhynchus mykiss*) fry reared under different levels of total suspended solids in the water after 56 days.

	RAS	BFT250	BFT350	*p* Values
TAC (μM)	902.18 ± 130.37	960.87 ± 192.66	923.36 ± 157.89	0.072
GR (nmol·min^−1^·mg prot^−1^)	225.07 ± 39.61	292.29 ± 125.46	233.09 ± 36.92	0.593
SOD (%)	56.57 ± 12.48	59.02 ± 6.19	65.14 ± 7.89	0.144
GPx (nmol·min^−1^·mg prot^−1^)	121.67 ± 42.99	99.76 ± 33.40	103.07 ± 26.38	0.392

Means do not differ by the ANOVA test (*p* > 0.05). RAS: recirculation aquaculture system; BFT250: biofloc 250 mg L^−1^; BFT350: biofloc 350 mg L^−1^. TAC—total antioxidant capacity; GPx—total glutathione peroxidase; GR—glutathione reductase; SOD—superoxide dismutase.

## Data Availability

The data that supports the findings of this study are available from the corresponding author upon request.
